# Patient involvement in the encounter between general practice and patients with a chronic disease. Results of a scoping review focusing on type 2 diabetes and obstructive pulmonary disease

**DOI:** 10.1080/13814788.2022.2153827

**Published:** 2022-12-12

**Authors:** Signe Beck Titlestad, Michael Marcussen, Marie Sandstød Rasmussen, Birgitte Nørgaard

**Affiliations:** Department of Public Health, University of Southern Denmark, Odense, Denmark

**Keywords:** Patient involvement, general practice, COPD, TD2, patient perspectives, GP’s perspective

## Abstract

**Introduction:**

Research has shown improved health outcomes when patients are involved in managing their health conditions and when their individual needs are considered.

**Objectives:**

This scoping review aimed to map the existing research regarding chronic disease patients’ involvement in their encounters with general practice, with a specific focus on patients with Type 2 diabetes (TD2) or chronic obstructive pulmonary disease (COPD) and from the perspectives of both general practitioners and patients.

**Methods:**

Studies of any design, date, and language were included. A systematic search was conducted using the following databases: Medline, CINAHL, PsycInfo, Scopus, and EMBASE from August until October 2020 and renewed September 2021. Data were systematically charted by the following study characteristics: bibliographic aims; study aims; setting; area of interest; results; conclusion.

**Results:**

Eighteen studies were included; they conducted qualitative methods, surveys or mixed methods. From the patients’ perspectives, the importance of being more involved in treatment discussions during consultations as well as a friendly environment, was underscored. A good relationship and relational continuity make it easier for patients to be more involved in treatment decisions. From the general practitioner (GP) perspectives, they mentioned their high workload, long-standing relationships, knowledge about the patients and prepared patients as factors influencing their ability to involve patients in treatment discussions.

**Conclusion:**

A good GP–patient relationship was considered an important aspect to providing and facilitating for involvement of patients with COPD or TD2.

Scoping review registration: https://osf.io/ynqt2

KEY MESSAGES

- Both patients and GPs underscore the importance of a good GP–patient relationship for facilitating involvement of patients with COPD or TD2.
- Furthermore, balancing GPs’ professional responsibility of achieving the best health outcome for their patients and respecting patients’ health beliefs is a relevant factor in patient involvement.

## Introduction

Globally, there is an increasing interest in patient involvement in healthcare [1,2]. Multiple definitions of patient involvement exist and among those a definition by the British Medical Association: ‘*the active participation of citizens, users and careers and their representatives in the development of health care services and as partners in their own health care*’ [3]. Research demonstrates improved health outcomes when patients are involved in the management of their own health conditions and when their individual needs are taken into account [4]. Furthermore, the positive influence of patient involvement to treatment and well-being has been established in terms of increased satisfaction [5], higher compliance [6], patient safety [5,7], enhanced psychological well-being [8], improved clinical outcomes and reduced healthcare-system costs [6,9,10]. Moreover, involving patients in treatment decisions benefits healthcare professionals by enhancing their understanding of patients’ health problems, thus contributing to effective management of health delivery by gaining patients’ trust and by enabling them to deliver individualised and tailored healthcare [11].

Worldwide, the number of people living with chronic diseases is rising, and the burden of chronic diseases is increasing rapidly. Globally, approximately one in three adults suffer from one or more chronic diseases [12]. Chronically ill patients tend to have complex pathways characterised by multimorbidity and multiple contacts across the healthcare system. To meet this complexity, general practitioners (GPs) are increasingly given the primary treatment responsibility for patients with chronic disease [13]. Being gatekeepers and the primary contact to healthcare, GPs play an important role in chronically ill patients’ perception of being involved in their treatment and care. However, analyses of chronic diseases patients’ involvement in their encounters with general practice appear to be lacking. We chose to focus on chronic obstructive pulmonary disease (COPD) and type 2 diabetes (TD2) as they are the two most prevalent chronic conditions that are managed by GPs in Western countries [14,15]. In a situation where policymakers continue to push for the adoption of patient involvement in general practice of patients with COPD and TD2 [13], there is an urgent need to remedy the lack of knowledge in this field. This scoping review aimed to map the existing research regarding chronic disease patients’ involvement during their encounters with general practice, with a specific focus on patients with TD2 or obstructive pulmonary disease and from the perspectives of both general practitioners and patients.

## Method

### Protocol and registration

This scoping review is reported according to the Preferred Reporting Items for Systematic Reviews and Meta-Analyses Extension for Scoping Reviews (Prisma-ScR) [16]. The protocol was registered in the Open Science Framework (osf.io/ynqt2) and remained unchanged during the review.

### Eligibility criteria

Studies reporting the involvement of adult patients aged 18 years or older and with TD2 or COPD were included. The core concept examined by this scoping review is patient involvement, defined as active participation in healthcare decision-making, patient involvement in healthcare consultation, factors associated with patient involvement and patients’ preference for involvement. The context is limited to general practice and their encounters with GPs. Studies reporting on patients with mental illness, patients in palliative care, and studies reporting results from multiple diagnoses, populations or age groups and where the results were not stratified were excluded. Studies reporting patient involvement with other healthcare professionals in general practice were excluded. If studies included patients with different diagnoses, patients both over and under 18 years of age, the studies were considered for inclusion only if data was stratified. Similarly, if studies included both a patient perspective and the perspectives of GPs or other healthcare professionals, the studies were included only if the patients and GPs perspectives were presented separately. Literature of any date and language was included.

### Information sources and search strategy

Both systematic and explorative literature searches were used to uncover the field of research in the best possible manner. A systematic search was conducted using the following databases: Medline, CINAHL, PsycInfo, Scopus and EMBASE. All databases were searched from August 2020 until October 2020. The search was renewed on 10 September 2021.

The search was performed by the first author and guided by an information specialist. Validated and pre-tested search filters were used as inspiration for developing our matrix [17,18]. The search strategy included subject indexing terms and free-text terms for title, abstract, and keyword searching. The search terms were grouped into three concepts and arranged per the PCC (population, concept, context) framework as recommended for scoping reviews (Table 1) [19]. The full version of the search is documented as supplementary material. No restrictions were placed in terms of publication year or country. Simultaneously, an explorative chain search was conducted using reference and citation analyses of the publications identified by the systematic search. In addition, reference lists of included studies were screened for relevant articles, and experts’ literature and libraries were also screened.

**Table 1.** Search matrix.

Population: patients with chronic disease	Concept: patient involvement	Context: general practice
Chronic disease (MeSH)	Patient participation (MeSH)	General practitioners (MeSH)
Diabetes, Mellitus type 2 (MeSH)	Decision Making (MeSH)	Physicians, Family(MeSH)
Pulmonary disease, Chronic obstructive (MeSH)	Clinical Decision-Making	General practice (MeSH)
	Decision Making, shared (MeSH)	Primary health care (MeSH)
	Self-Management (MeSH)	Family practice (MeSH)
	Empowerment (MeSH)	
	Patient care planning (MeSH)	
	Self-care (MeSH)	
	Patient compliance (MeSh)	
	Cooperative behaviour (MeSh)	
	Physician-patient relations (MeSh)	
Free text		
(Chronic disease*) or (Chronic disorder) or (Chronic illness*) or (Diabetes type 2) or (T2D) or (COPD) or (Chronic pulmonary obstructive disease*) or (long-term condition*)	((activati* or participat* or involv* or engag* or influenc* or impact or perspective* or collaborat* or contribut* or adherence or centred or includ* or inclusion or voice* or view* or intergra* or led or partner*) adj2 (patient or client or user or consumer))	(General practi*) or (family practi*) or (family physician*) or (primary care) or (GP)
		
	Or	
	((Co-production adj2 knowledge) or shared decision making or self-management or empowerment or partnership)	

### Study selection

All identified studies were uploaded to Endnote (https://endnote.com/). Doublets were removed prior to importation to the reference programme Covidence.org. Before study selection, the predefined eligibility criteria were pilot-tested in a sample of 50 articles (SBT, MM, and BN). Two authors (SBT and BN) independently screened the remaining titles and abstracts, and disagreements were resolved through discussion or via the involvement of a third author (MM). Eligibility does not necessarily have to be established before the literature search in a scoping review; eligibility criteria can be developed as the knowledge of the identified literature grows [20]; accordingly, the eligibility criteria for this scoping review were adjusted after screening the titles.

### Data extraction

Two reviewers (SBT, BN) performed data extraction independently using predefined data-extraction spreadsheets. Discrepancies were negotiated until consensus was reached, with a third reviewer (MM) available to resolve conflicts. Data were systematically charted by the following study characteristics: Bibliographic aims; study aims; setting; area of interest; results; conclusion.

### Data synthesis

The included studies’ key findings were descriptively summarised and narratively presented. A textual narrative synthesis was applied as described by Lucas et al. This approach comprises a commentary reporting of study characteristics, context, quality, and findings based on differences and similarities among studies [21]. Thus, the extracted studies were first grouped by the perspectives reported (i.e. perspectives of general practitioners and patients with TD2 or COPD). Afterwards, patterns across the studies were identified and presented descriptively and narratively.

## Results

### Study selection

**Figure 1.**
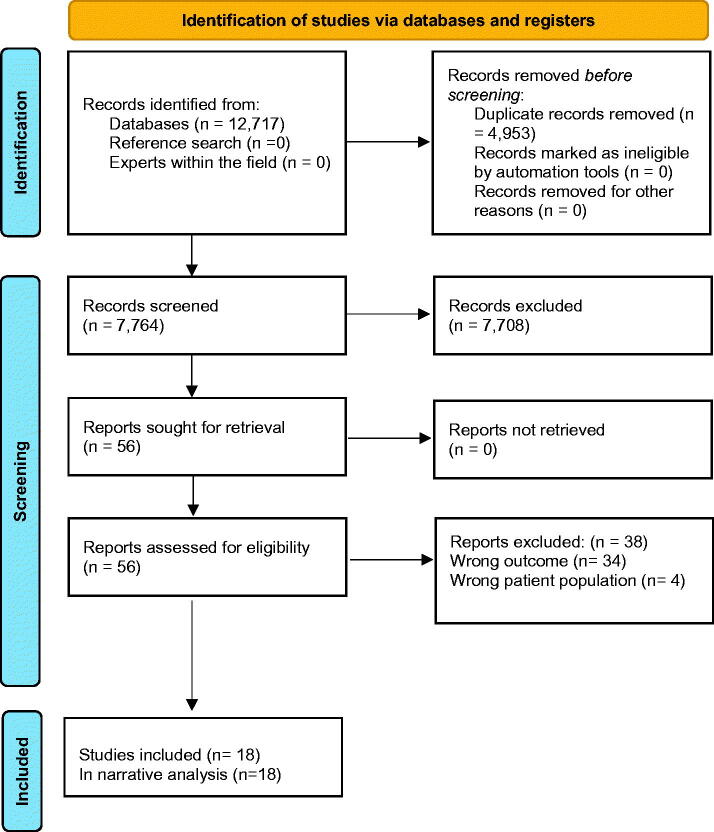
Prisma flow diagram.

We identified a total of 12,717 studies. After de-duplication, 7764 title abstracts were screened, and 7708 citations were excluded due to wrong outcomes or wrong population, leaving 56 studies for full-text reading, of which 18 studies met the inclusion criteria. The process is illustrated in Figure 1.

### Description of included studies

The included studies were published between 2001 and 2020. Four of the 18 studies were undertaken in Australia [22–25], three in the USA [26–28], two in Sweden [29,30], Norway [31,32], UK [33,34], New Zealand [35,36], and Belgium [37,38], and one in Malaysia [39]. For data collection, semi-structured interviews, focus group interviews, telephone interviews, video recordings, and observations were used. Furthermore, the mixed-methods studies included questionnaires, interviews, and observations. The most frequent investigative methods were content [22,29–31] or thematic [23,25,32,34,38] analysis, whereas one study applied a grounded theory approach [28]. Of the included studies, there was an underrepresentation of studies, including patients with COPD because no studies contained knowledge on the involvement of patients with COPD from the patients’ perspectives and only two from GPs’ perspectives [32,36]. The remaining studies reported on the involvement of patients with TD2 from either the patients’ [22,23,26,29,31,33,38] or GPs’ [24,30,32,36,37] views or a combination thereof [25,27,28,34,35,39]. In Table 2, the included studies’ characteristics are presented. The studies comprised 1132 participants (843 patients and 289 GPs), with 10–322 patients with TD2 per study and 4–67 GPs. Most studies applied purposive or randomly selected sampling strategies.

**Table 2.** Study characteristics.

Author, year of publication, location	Aim	Study design	Inclusion and exclusion criteria	Design/data collection method	Sampling strategy	Participants’ characteristics (Gender, Age)	Data analysis techniques
Abdulhadi, 2007 (Sweden)	To explore the perceptions of TD2 patients regarding the medical encounters and quality of interactions with their primary healthcare providers.	Qualitative	Patients with TD2 (*n* = 27)	Focus group interview	Purposive sampling	13 men and 14 women Age range 26–70 years	Qualitative content analysis
Abdulhadi, 2013, (Sweden)	To explore the experiences of primary healthcare providers of their encounters with patients with TD2, and their preferences and suggestions for future improvement of diabetes care	Qualitative	General practitioners (*n* = 19)	Semi-structured interview	Purposive sampling	8 women, 11 men, Age range 29–55 years	Qualitative content analysis
Ball, 2016 (Australia)	To examine the perceptions of patients who have been recently diagnosed with TD2 regarding nutrition care provided by primary healthcare professionals	Qualitative	Patients with TD2 (*n* = 10)	Semi-structured telephone interviews	Purposive sampling	3 men, 7 women, Age range 27–74 years.	Content analysis and meta-synthesis of findings over time
Burridge, 2016 (Australia)	Patients’ perceptions and experiences of TD2 selfcare and engagement with GP-led integrated diabetes care	Qualitative	Patients with TD2 (*n* = 30)	Interviews	Purposive sampling	14 women and 16 men. Mean age: 60.2	Thematic analysis
Dambha-Miller, 2018 (UK)	To explore patients’ views on factors within patient-practitioner interactions that are of significance to them after diagnosis and over a 10-year experience of living with the disease	Mixed methods	Patients with TD2 (*n* = 311) 1 year follow-up. (*n* = 110) 10 year follow-up. (*n* = 46) both times.	Survey – Free text comments	Not described	1 year follow up: 196 men/142 women. Mean age 60 years old. 10 year follow up. 53 men/48 women. Mean age 72 years old.	Descriptive analysis. Cross-sectionally analysis
Dao, 2019 (Australia)	To explore the factors influencing self-management of TD2 in patients attending general practice in southwest Sydney from both a patient and provider perspective. A secondary aim was to assess how consistent the findings were with the socio-ecological model	Qualitative	Patients with TD2 (*n* = 10) General practitioner (*n* = 4)	Semi-structured interviews	Purposive sampling	Patients: 6 women and 4 men. Age range 30–79. GP: 6 women, and 4 men. Age range 30–79.	Thematic analysis
Dowell, 2018 (New Zealand)	To observe in detail the primary care interactions and communications of patients with newly diagnosed diabetes over time. In addition to identify key points on the process where miscommunication might occur	Qualitative	Patients with TD2 (*n* = 32) General practitioner (*n* = ?)	Video Recording	Purposive sampling	Not described	Ethnography and interaction analysis
Grant, 2016 (USA)	To examine how patients with TD2 and their primary care physicians identify and discuss visits prior to and during visits.	Qualitative	Patients with TD2 (*n* = 29) General practitioner (*n* = 67)	Qualitative interviews and focus group	Purposive sampling	Patients: Age range 35–80) 14 women and 15 men. Primary care providers (61%) women.	Modified grounded theory approach with an inductive approach
Halliwell, 2016 (New Zealand)	To identify strategies that general practitioners can use to facilitate discussions of prognosis with patients who have COPD	Qualitative	General practitioner (*n* = 15)	Telephone interviews	Purposive sampling	7 women and 8 men. Age range 31–60	General inductive approach
Heisler, 2003 (USA)	To assess the extent to which patients with TD2 agree with their primary care provider on diabetes treatment goals and strategies, the factors that predict agreement and whether greater agreement is associated with better patient self-management of diabetes	Survey	Patients with TD2 (*n* = 123) General practitioner (*n* = 50)	Survey	Randomly sample	Patients: Mean age 65, 81% male. Providers mean age 40, 56% men	Descriptive statistics
Laue, 2016 (Norway)	To explore the decision-making of general practitioners concerning treatment with antibiotics and/or oral corticosteroids and hospitalisation for COPD patients with exacerbations	Qualitative	General practitioner (*n* = 53)	Focus group interview	Purposive sampling	Not described	Thematic analysis
Oftedal, 2020 (Norway)	How adults with TD2 perceive different attributes of support provided by healthcare practitioners and how various attributes of support can influence peoples’ motivation to self-manage their diseases	Qualitative	Patients with TD2 (*n* = 19)	Focus group interviews	Purposive sampling	12 men, 7 women. Median age 54, 52, 42	Content analysis
Parchman, 2010 (USA)	To examine a causal model linking participatory decision making to improved clinical outcomes that included patient activation and medication adherence	Mixed methods	Patients withTD2 (*n* = 144)	Observations and questionnaires	5 independent primary care practices	Mean age 57.7 years. 61% women and 39% mean.	Path analysis using a structural equation model
Pooley, 2001 (UK)	To explore people with TD2 and healthcare professionals who deliver their diabetes care. To explore the issues that they perceive as central to effective management of diabetes, primarily within a primary care setting	Qualitative	Patients with TD2 (*n* = 47) General practitioner (*n* = 7)	Focus group interviews	Randomly selected	Not described	Qualitative (thematic) analysis
Shortus, 2013 (Australia)	To investigate provider perspectives on the role of patient involvement in chronic disease decision making	Qualitative	General practitioner (*n* = 19) (+ 8 allied health providers, and 2 endocrinologists)	Interviews	Purposive sampling	Not described	Grounded theory
Syed, 2017 (Malaysia)	To investigate whether the use of a patient decision aid for insulin initiation fulfils its purpose of facilitating patient-centred decision making through identifying how doctors and patients interact when using the PDA during Primary care consultations	Qualitative	Patients with TD2 (*n* = 7) General practitioner (*n* = 7)	Audio- or video-recorded consultations (*n* = 15)	Purposive sampling	GPs: 1 man, 6 women Patients: 2 men, 5 women; age range 50–73)	Conversation analysis
Vermeire, 2003 (Belgium)	To examine the health beliefs of people living with TD2, the way they communicate about it, and the problem they encounter in adhering to therapeutic regimens	Qualitative	Patients with TD2 (*n* = 46)	Focus group interviews and observations	Focus groups divided by gender.	21 men, 25 women. Age range 40–80.	Thematic analysis
Wens, 2005 (Belgium)	To examine explicitly the physicians’ expectations of their diabetes patient compliance/adherence. Objectives: 1) elicit problems physicians encounter with TD2 diabetes patients’ adherence to treatment recommendations; 2) to search for solutions and 3) to discover escape mechanisms in case of frustration	Qualitative	General practitioners (*n* = 40)	Focus group interviews and observations	By interest	26 women, 14 men. Mean age 45.3. Mean years in practice 18.4	Content analysis

### Narrative synthesis

#### Patients’ perspectives

Patients reported the importance of GPs providing them with sufficient time for consultations and explaining or discussing things with them. It was essential that the GPs were not too rushed and preoccupied with their own agendas under time-pressure constraints [22,23,28,33,34]. The lack of time to deal effectively with patients’ concerns and the beliefs among the patients were repeatedly mentioned [34]. Similarly, the patients stated that an unfriendly environment with poor attention and lack of eye contact with the GP prevented them from asking questions and expressing their concerns during consultations and made the encounters more doctor-centred [29]. The relational continuity of care was important to the patients, who preferred to see the same GP at each consultation. The patients expressed that they would benefit from the continuity in terms of increased familiarity with their circumstances [23,29,33,34]. In addition, the patients considered it problematic if they did not see the same GP every time because seeing the same GP would make it easier for them to discuss their problems and be more involved in treatment decisions [33]. The patients experienced that GPs differ in behaviour and methods of providing care and information, and the patients preferred to build a relationship with their GP to increase their ability to be more active in treatment decision-making [29]. This relationship was considered a partnership defined as when the competence of the GPs and the patients’ knowledge complemented each other [23,28,31,34,38]. The patients wanted to cooperate with their GP but found it highly difficult if their needs were not listened to [31]. Furthermore, patients reported that their GPs paid too little attention to their health beliefs and the opinions and concepts they held on medicine in general, their illness, and medicine-taking [38]. Patients identified key factors associated with more adequate discussion of their visit’s priorities, including GPs’ willingness to be flexible in the flow of discussion topics and the extent to which their GPs listened to their concerns [28]. It was evident that an active patient/provider alliance was important for engaging the patients in the work of disease management but likely also a critical factor in sustaining the long-term collective work that diabetes management requires [23]. Low literacy was mentioned as a barrier to being involved in treatment decisions because the patients believed that they had to accept what has been demonstrated to them [29]. However, identifying visits’ priorities in advance enhanced a proactive and collaborative approach, made it possible for the patients to bring what they wanted to discuss, and did not prevent the GPs from talking about what they wanted to express. Thus, a personalised approach offered by GPs was valued and the patients expressed a wish that the GPs should be better at listening in a more holistic approach [23,31].

#### General practitioners’ perspectives

GPs mentioned high workload as a major problem affecting their interactions with the patients because building good relationships with patients takes time [25,30]. GPs expressed the negative impact of time constraints on the ideal goal of entirely electing patient priorities; being in a rush, they often had their own agenda overriding the patient’s agendas [28]. A good relationship with patients was considered necessary in relation to involving them in treatment decisions [25,30,32,36,37]. The GPs’ knowledge about their patients appeared to be important in assessing whether they could rely on the patient’s own judgement about the necessity for treatment and hospitalisation. They believed the patients were good at assessing their needs themselves [32]. Some GPs suggested that there should be a personal interest of healthcare providers in care and to show interest to the patients. Furthermore, they proposed avoiding giving instructions to the patients but, rather, having good communication and respecting their concerns; such an approach would be a more helpful way to correct the patients’ understanding of their disease and gain their cooperation [30]. The GPs indicated that they favoured active involvement of the patient’s experiential knowledge due to having lived with the disease not necessarily reflecting a biomedical need but also the requirement for relieving anxiety and meeting challenges in the patients’ social lives [32,35]. Consequently, there could be discrepancies between the GPs’ and patients’ judgments, which the GPs had to balance. According to the GPs, a lack of patients’ consent to treatment or hospital referral seemed particularly challenging [32].

GPs noted that a tool to empower patients to help them formulate their top priorities at consultations would be helpful, and the GPs liked the idea of pre-visit preparation [28]. The GPs acknowledged that there should be potential for flexibility in the application of guidelines and that involving patients in decision-making was an important consideration in delivering high-quality care. However, they believed that it was more caring to insist upon treating a patient’s disease than to respect any patient preference and that they should endeavour to persuade patients to accept their advice. The GPs saw their primary responsibility as doing whatever was necessary to minimise the possibility of achieving less-than-ideal outcomes. GPs often experienced a conflict between the two professional responsibilities of achieving ideal health outcomes for their patients and respecting their patients’ rights to make decisions where compromise is often necessary [32].

## Discussion

### Main findings

Across studies and perspectives, our findings indicate that time at consultation, relational continuity, and a good GP–patient relationship are important for providing and facilitating patient involvement. These findings align with research showing that continuity in care, a supportive consultation environment with a warm and caring GP, and a good patient–GP interaction is significant in chronic disease management [14,37].

### Patients’ perspectives

For the patients, their GP must pay attention to their health beliefs, opinions and concepts they hold about their treatments, illnesses, and medicines, corroborating findings from Kennedy et al. who found that healthcare providers’ understanding of their patients’ healthcare beliefs, values, and preferences is considered important by both patients and healthcare providers. If these providers listen and communicate with patients, they are more likely to develop a shared understanding that may improve future decision-making [40]. This approach is also supported by McMillan [41], who found that the patients’ beliefs and opinions regarding their disease and treatment affect their self-management. Exploring patients’ beliefs and values is crucial to understanding how to provide patient-centred care.

### GPs’ perspectives

However, in some of the included studies, the interviewed GPs experienced conflicts between their professional responsibilities of achieving what they consider ideal health outcomes for their patients and respecting their patients’ health beliefs and their rights to be involved in decisions. Abu Hassan et al. found that exploring patients’ concerns and beliefs to treatment is crucial to assist physicians in delivering patient-centred care because negative concerns and beliefs towards treatments or decisions made by the physician might cause barriers to accepting the treatment [42]. Thus, patients’ knowledge and health beliefs and GPs personal knowledge about a specific patient should be acknowledged as a conceptual resource for patient involvement, but how the GPs should balance their own and patients’ preferences should be explored further. However, these aspects apply mainly to patients with TD2 and their GPs because no studies were found reporting on patients with COPD. Only two studies reported on GPs’ perspectives on the involvement of patients with COPD. This literature gap is of concern, given the association between stigma-related experiences and essential patient outcomes and the fact that there are many unmet healthcare needs among patients with COPD [43]. Furthermore, research shows that many people with lung disease have faced stigma due to their condition and research lacks awareness of this aspect [44]. Furthermore, patients with lung disease tend to feel isolated, which makes it even more important to engage patients with COPD to be more involved in treatment decisions [45]. Thus, the included studies indicate that focus and action must be directed towards research exploring patients with COPD perspectives on patient involvement in general practice.

### Strengths and limitations

This study was designed and reported in line with the recommendations of the PRISMA-ScR statement [16]. Multiple databases were searched, and a thorough search strategy was designed iteratively by the research team and an information specialist to account for the three different dimensions of the search (patients with TD2 or COPD, patients’ involvement, and general practice). All aspects of data collection, extraction, and analysis were carried out independently by two researchers, with a third party available for mediation in cases of disagreements. The primary limitation of this scoping review is the sparse literature related to our objectives that may be caused by choice to focus on chronic obstructive pulmonary disease (COPD) and type 2 diabetes (TD2) as they are the two most prevalent chronic conditions that are managed by GPs in Western countries [14,15,46–48]. On the other hand, no results were found to suggest that the perspectives of patients with COPD and type 2 diabetes regarding involvement should differ considerably from those of patients suffering from other chronic conditions. The quality of the included studies was not assessed because this is not typically a part of a scoping review due to an effort to maintain a broad perspective and include studies with different methods and designs.

## Conclusion

A good GP–patient relationship was considered an important aspect in relation to providing and facilitating for involvement of patients with COPD or TD2. Furthermore, conflicts between GPs’ professional responsibility of achieving the best health outcome for their patients and respecting patients’ health beliefs are relevant factors in patient involvement. The lack of studies investigating patient involvement in general practice of patients with COPD or TD2 may suggest the need for further research. Our findings indicate that the need for future research is vital for patients with COPD.

## Supplementary Material

PRISMA-ScR-Checklist

Supplemental Material: The search strategies applied for the four databases

## Data Availability

Full search string available as supplementary material.
