# Supplementary material for: Patient involvement in the encounter between general practice and patients with a chronic disease. Results of a scoping review focusing on type 2 diabetes and obstructive pulmonary disease
Source: Eur J Gen Pract. 2022 Dec 12;28(1):260–9. doi: 10.1080/13814788.2022.2153827 (PMC9754033; doi:10.1080/13814788.2022.2153827)
Supplement: Supplemental Material: The search strategies applied for the four databases [file IGEN_A_2153827_SM2158.docx]

Appendix 1

*The search strategies applied for the four databases*

**Medline**

September 2021

| 1 | Chronic Disease/ |
| --- | --- |
| 2 | Diabetes Mellitus, Type 2/ |
| 3 | Pulmonary Disease, Chronic Obstructive/ |
| 4 | 1 or 2 or 3 |
| 5 | (Chronic disease* or Chronic disorder or Chronic illness* or Diabetes type 2 orCOPD or Chronic pulmonary obstructive disease* or long-term condition*) |
| 6 | 4 or 5 |
| 7 | Patient Participation/ |
| 8 | Decision Making/ |
| 9 | Clinical Decision-Making/ |
| 10 | Self-Management/ |
| 11 | Empowerment/ |
| 12 | Patient Care Planning/ |
| 13 | Self Care/ |
| 14 | Patient Compliance/ |
| 15 | Cooperative Behavior/ |
| 16 | Physician-Patient Relations/ |
| 17 | Cooperative Behavior/ |
| 18 | "Treatment Adherence and Compliance"/ |
| 19 | 7 or 8 or 9 or 10 or 11 or 12 or 13 or 14 or 15 or 16 or 17 |
| 20 | ((activati* or participat* or involv* or engag* or influenc* or impact or  perspective* or collaborat* or contribut* or adherence or centered or includ* or  inclusion or voice* or view* or intergra* or led or partner*) adj3 (patient or client  or user or consumer)) |
| 21 | ((Co-production adj2 knowledge) or shared decision making or selfmanagement  or empowerment or partnership) |
| 22 | 19 or 20 |
| 23 | 18 or 21 |
| 24 | General Practitioners/ |
| 25 | Physicians, Family/ |
| 26 | General Practice/ |
| 27 | Primary Health Care/ |
| 28 | Family Practice/ |
| 29 | 23 or 24 or 25 or 26 or 27 |
| 30 | (General practi* or family practi* or family physician* or primary care or  GP). |
| 31 | 28 or 29 |
| 32 | 6 and 22 and 30 |

**Psycinfo**

September 2021

| 1 | Chronic Illness/ |
| --- | --- |
| 2 | Chronic Obstructive Pulmonary Disease/ |
| 3 | Type 2 Diabetes/ |
| 4 | 1 or 2 or 3 |
| 5 | (Chronic disease* or Chronic disorder or Chronic illness* or Diabetes type 2 orCOPD or Chronic pulmonary obstructive disease* or long-term condition*) |
| 6 | 1 or 2 or 3 or 4 or 5 |
| 7 | Client Participation/ |
| 8 | Self-Care Skills/ |
| 9 | Decision Making/ |
| 10 | Self-Management/ |
| 11 | Empowerment/ |
| 12 | Treatment Compliance/ |
| 13 | Collaboration/ |
| 14 | Client Education/ |
| 15 | 7 or 8 or 9 or 10 or 11 or 12 or 13 or 14 or 15 or 16 |
| 16 | ((activati* or participat* or involv* or engag* or influenc* or impact or  perspective* or collaborat* or contribut* or adherence or centered or includ* or  inclusion or voice* or view* or intergra* or led or partner*) adj3 (patient or client  or user or consumer)) |
| 17 | ((Co-production adj2 knowledge) or shared decision making or selfmanagement  or empowerment or partnership). |
| 18 | 18 or 19 |
| 19 | 17 or 20 |
| 20 | Family Physicians/ |
| 21 | General Practitioners/ |
| 22 | Primary Health Care/ |
| 23 | 22 or 23 or 24 |
| 24 | (General practi* or family practi* or family physician* or primary care or  GP) |
| 25 | 25 or 26 |
| 26 | 6 and 21 and 27 |

**EMBASE**

September 2021

| 1 | Chronic Disease/ |
| --- | --- |
| 2 | non insulin dependent diabetes mellitus/ |
| 3 | chronic obstructive lung disease/ |
| 4 | 1 or 2 or 3 |
| 5 | (Chronic disease* or Chronic disorder or Chronic illness* or Diabetes type 2 orCOPD or Chronic pulmonary obstructive disease* or long-term condition*) |
| 6 | 4 or 5 |
| 7 | Patient Participation/ |
| 8 | Decision Making/ |
| 9 | Clinical Decision-Making/ |
| 10 | patient decision making/ |
| 11 | shared decision making/ |
| 12 | self care/ |
| 13 | empowerment/ |
| 14 | patient care planning/ |
| 15 | teamwork/ |
| 16 | doctor patient relationship/ |
| 17 | 7 or 8 or 9 or 10 or 11 or 12 or 13 or 14 or 15 or 16 or 17 |
| 18 | ((activati* or participat* or involv* or engag* or influenc* or impact or  perspective* or collaborat* or contribut* or adherence or centered or includ* or  inclusion or voice* or view* or intergra* or led or partner*) adj3 (patient or client  or user or consumer)). |
| 19 | ((Co-production adj2 knowledge) or shared decision making or selfmanagement  or empowerment or partnership). |
| 20 | 19 or 20 |
| 21 | 18 or 21 |
| 22 | general practice/ |
| 23 | general practitioner/ |
| 24 | primary health care/ |
| 25 | 23 or 24 or 25 |
| 26 | (General practi* or family practi* or family physician* or primary care or  GP). |
| 27 | 26 or 27 |
| 28 | 6 and 22 and 28 |

**Cinahl**

September 2021

| 1 | MH "Chronic Disease") |
| --- | --- |
| 2 | MH "Pulmonary Disease,Chronic Obstructive") |
| 3 | MH "Diabetes Mellitus, Type 2") |
| 4 | S1 OR S2 OR S3 |
| 5 | (Chronic disease* or Chronic disorder or Chronic illness* or Diabetes type 2 orCOPD or Chronic pulmonary obstructive disease* or long-term condition*) |
| 6 | S4 OR S5) |
| 7 | MH "Consumer Participation") |
| 8 | (MH "Decision Making") |
| 9 | MH "Decision Making, Shared") |
| 10 | MH "Decision Making, Patient") |
| 11 | (MH "Self Care") |
| 12 | (MH "Empowerment") |
| 13 | (MH "Patient Care Plans") |
| 14 | (MH "Patient Compliance") |
| 15 | (MH "Patient Centered Care") |
| 16 | (MH "Professional-Patient  Relations") |
| 17 | MH "Coalition") |
| 18 | MH "Cooperative Behavior") |
| 19 | S7 OR S8 OR S9 OR S10 OR S12 OR S13 OR S14 OR S15 OR S16 OR S17 OR S18 |
| 20 | ((Co-production adj2 knowledge) or shared decision making or selfmanagement  or empowerment or partnership). |
| 21 | ((activati* or participat* or involv* or engag* or influenc* or impact or  perspective* or collaborat* or contribut* or adherence or centered or includ* or  inclusion or voice* or view* or intergra* or led or partner*) adj3 (patient or client  or user or consumer)). |
| 22 | S20 OR S21 |
| 23 | S19 OR S22 |
| 24 | MH "Physicians, Family") |
| 25 | (MH "Family Practice") |
| 26 | (MH "Primary Health Care") |
| 27 | S24 OR S25 OR S26 |
| 28 | (General practi* or family practi* or family physician* or primary care or  GP).” |
| 29 | S27 OR S28 |
| 30 | S27 OR S28) AND (S6 AND S23 AND S29) |
